# Sleep strengthens successor representations of learned sequences in humans

**DOI:** 10.1371/journal.pbio.3003740

**Published:** 2026-04-07

**Authors:** Xianhui He, Philipp K. Büchel, Simon Faghel-Soubeyrand, Janina Klingspohr, Marcel S. Kehl, Bernhard P. Staresina

**Affiliations:** 1 Department of Experimental Psychology, University of Oxford, Oxford, United Kingdom; 2 Department of Epileptology, University Hospital Bonn, Venusberg Campus, Bonn, Germany; 3 Department of Systems Neuroscience, Universitaetsklinikum Hamburg Eppendorf, Hamburg, Germany; 4 Oxford Centre for Human Brain Activity, Centre for Integrative Neuroimaging, Department of Psychiatry, University of Oxford, Oxford, United Kingdom; International School for Advanced Studies, ITALY

## Abstract

Experiences reshape our internal representations of the world. However, the neural and cognitive dynamics of this process are largely unknown. Here, we investigated how sequence learning reorganizes neural representations and how sleep-related consolidation mechanisms contribute to this transformation. Using high-density electroencephalography and multivariate decoding, we found that learning temporal sequences of visual information led to the incorporation of successor representations during a subsequent perceptual task, despite temporal information being task-irrelevant. Importantly, individuals with better sequence memory performance exhibited stronger successor incorporation during the perceptual task. Representational similarity analyses comparing neural patterns with different layers of a deep neural network revealed a learning-induced shift in representational format, from low-level visual features to higher-level abstract properties. Critically, both the strength and transformation of successor representations correlated with the neurophysiological hallmarks of slow-wave sleep during a post-learning nap, particularly the coupling between slow oscillations and spindles. These findings support the idea that sequence learning induces lasting changes in visual representational geometry and that sleep physiology strengthens these changes, providing mechanistic insights into how the brain updates internal models after exposure to environmental regularities.

## Abstract 2

The neural dynamics of how experiences reshape our internal representations of the world are largely unknown. This study shows that learning sequential experiences reshapes how the brain represents what we see and that a post-learning nap strengthens these predictive changes.

## Introduction

**Fig 1:**
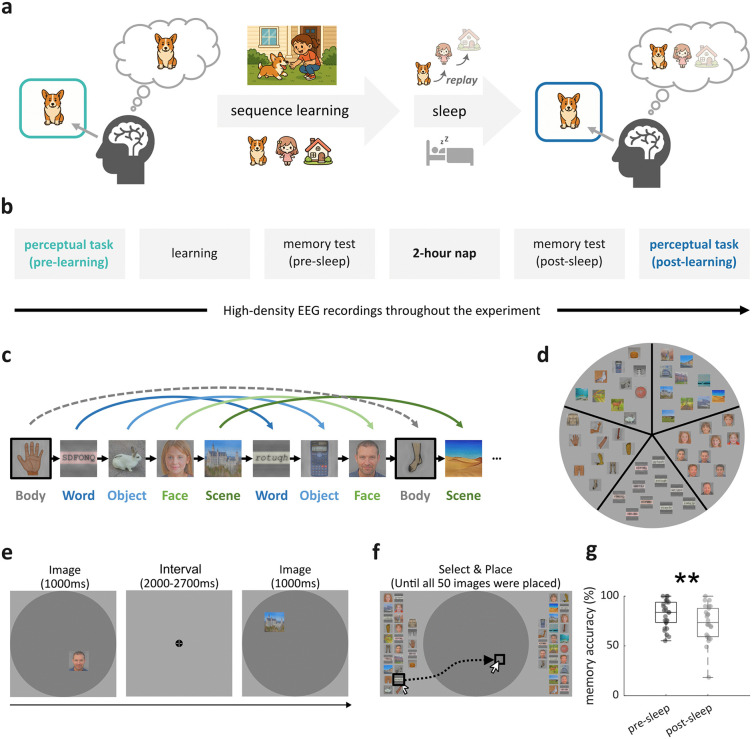
Emergence of Successor Representations and Experimental Design. **(a)** Example of how sequence learning and sleep might change neural representations. Upon encountering a Welsh Corgi, the brain primarily represents the current stimulus entity. If the Corgi is part of a recurring temporal sequence (Corgi → Girl → House), subsequent stimuli (Girl and House) might be integrated into the Corgi representation. Post-learning sleep might provide an opportunity for the brain to replay learned experiences and thereby further strengthen successor representations. Upon post-sleep exposure to a Corgi image, brain activation patterns might reflect both the current stimulus (Corgi) as well as learned successors (Girl, House). Faded images indicate weaker representations; **(b)** Timeline of the experiment. Participants first completed a perceptual task, followed by a sequence learning task (Memory Arena). Memory for the learned sequence was then assessed both before and after a period of sleep. Finally, participants completed the perceptual task again; **(c)** Memory Arena sequence design. Participants (*N* = 26) were tasked with learning the spatiotemporal structure of 50 images. These images belonged to five distinct categories (letter strings, scenes, objects, faces, and body parts) and were organized into 10 subsequences of five images each, following one of two fixed category orders: (i) letter string, scene, object, face, or (ii) object, scene, letter string, face, with body part images randomly inserted to obscure the primary category sequences. The two subsequence types were counterbalanced across participants; **(d)** Memory Arena location design. The Arena was spatially organized into five principal ‘slices’, with each slice corresponding to one of the five main image categories; **(e)** Example learning trial. Images were presented sequentially at their unique positions. Participants were asked to learn the image and its location; **(f)** Example test trial. All 50 images were randomly distributed around the Arena. Participants selected each image by clicking on it and then click exact position within the arena where they remembered the image had been located; **(g)** Memory accuracy in pre- and post-sleep test. Each dot represents one participant. ** indicates significant difference in memory accuracy between pre- and post-sleep test (*p* = 0.002, *N* = 26, two-tailed paired *t* test). Icons originally generated using AI tools and the experimental stimuli have been replaced with materials from open-source resources (https://commons.wikimedia.org, https://openclipart.org/) and an AI-generated face dataset (https://github.com/RichardErkhov/ai_generated_faces), used under the MIT License.

External experiences continuously shape and update the brain’s internal model of the world. These experiences are dynamic though, and individuals do not merely encode representations of isolated events but also establish structured relationships between them. For example, a child who sees a Welsh Corgi for the first time may encode only a basic representation of a “small, fluffy creature.” However, upon repeatedly observing the Corgi following a girl into a house, the child might begin to associate these elements, gradually constructing a predictive model of their temporal co-occurrence (Fig 1a). While this example illustrates how we update our world model in daily life, the mechanisms underlying the integration of temporal regularities into existing representations remain poorly understood.

According to the ‘successor representation’ theory, the brain does not merely represent the current state of the world but also maintains a predictive map, encoding expected future states based on learned temporal transitions [1–3]. This predictive coding mechanism is thought to support flexible behavior and inference by integrating both immediate states and anticipated future contingencies. While initially developed within the domain of reinforcement learning, recent research suggests that successor representations may also underlie broader cognitive processes, including the organization of human episodic memory [4–6].

Empirical studies provide supporting evidence that learning temporal regularities across experiences shapes subsequent neural representational geometry, i.e., the organization of stimulus-related activity patterns in a high-dimensional neural state space [7–9]. Specifically, representations of temporally proximal stimuli tend to be more similar to one another than to temporally distant stimuli [4,5,10–13]. Notably, this effect can emerge after relatively few exposures [12], persists even when such regularities are irrelevant for current task demands [4], and correlates with the replay of neuronal activity in the hippocampus during task-free awake breaks [4]. While some work has reported forward-dominant predictive structure [13], other studies have not distinguished between representations of past and future information, instead demonstrating that hippocampal activity patterns exhibit greater similarity for temporally adjacent stimuli, regardless of sequence direction [4,5]. Recent work has further highlighted the bidirectional nature of these representations, showing that both past and future elements of a sequence are encoded [14]. This raises the question whether sequence learning specifically promotes prospective representations (consistent with the successor representation theory), and whether such changes in neural representations generalize to other contexts where the initial sequence is no longer behaviorally relevant.

The finding that wake replay strengthens successor representations [4], moreover, begs the question whether sleep, known to constitute a privileged time window for memory reactivation and replay [15–19], contributes to the incorporation of successor representations. In particular, the coupling between slow oscillations and spindles has been associated with memory consolidation and the reactivation of learning representations [20–23]. Interestingly, a recent study has shown that a night of sleep after a real-life sequential learning experience (guided art tour) selectively bolsters temporal order memory while memory for visual-perceptual features steadily declined over the course of up to one year later [24]. Importantly, this selective strengthening of temporal memory was predicted by the duration of slow-wave sleep (SWS) following learning and the extent of slow oscillation-spindle coupling [24]. However, it remains unclear whether similar mechanisms are at play when sequence learning yields the incorporation of successor representations.

In this study, we investigate whether sequence learning reshapes neural representations such that successor information is incorporated in a subsequent task context where the sequence is no longer behaviorally relevant (Fig 1b). Specifically, we address three key questions: (1) Does sequence learning reshape stimulus representations to incorporate successor information? (2) What is the representational format of successor representations (e.g., low-level perceptual versus high-level conceptual)? (3) (How) does post-learning sleep physiology (SO–spindle coupling) contribute to the incorporation of successor representations? To tackle these questions, we combined behavioral tasks, high-density electroencephalography (EEG) including Polysomnography and deep neural network (DNN)-based representational similarity analysis (RSA). Analytically, we operationalize representational geometry as the relational structure of multivariate stimulus-evoked activity patterns in neural response space, focusing on (i) the information that is linearly decodable from these patterns [8] and (ii) their pairwise (dis)similarity structure [25]. Specifically, we quantify successor-related decodability using multivariate classification and characterize learning-related changes in representational similarity structure using RSA aligned to hierarchical features from a deep neural network. Our findings revealed that successors of learned images could be reliably decoded in a subsequent non-memory task, with immediate post-learning memory performance predicting the strength of this effect. Additionally, DNN-based RSA suggested that successor representations shifted toward high-level visual features after learning. Importantly, greater SO–spindle coupling during SWS predicted the strength of subsequent successor representations as well as their shifts towards high-level formats. These results suggest that learning reshapes the representational geometry of visual experiences and that sleep physiology contributes to this transformation.

## Results

Participants first performed a perceptual task, in which 50 unique images from five categories (objects, faces, scenes, letter strings, and body parts; Fig 1c) were shown in pseudorandom order, with the instruction to press a button when a given image repeated from one presentation to the next (10% of “target” trials, “1-back” task). The perceptual task was followed by the “Memory Arena” task presenting the same images in fixed temporal sequences and spatial locations (Fig 1c–1f; [26]). Participants were instructed to memorize the identity and location of each individual image. The 50 images were organized into 10 subsequences of five images each, following one of two fixed category orders: (i) letter string, scene, object, face, or (ii) object, scene, letter string, face. Body part images were randomly inserted to obscure the primary category sequences (Fig 1c). Images from the same category were located in the same slice of the Arena (Fig 1d). Each learning cycle consisted of two exposure blocks, where images were presented sequentially at their respective locations (Fig 1e), followed by a test block in which participants reconstructed the sequence and spatial layout (Fig 1f). After reaching a learning criterion (66%; actual median sequence accuracy ± MAD: 79.5 ± 12.1%), participants took a ~ 2-hour nap. Memory accuracy was tested before and after sleep, followed by a repetition of the perceptual task. To mitigate possible ceiling effects and thereby render the task more sensitive to sleep-dependent consolidation [27], participants learned, in a single exposure-exposure-test round, an interference sequence with the same images but arranged into different subsequences that followed an alternative fixed category order (median sequence accuracy ± MAD: 16.3 ± 12.7%). By analyzing neural activity during the two perceptual tasks, we examined how successor representations changed with learning. Sleep EEG recordings allowed assessment of how sleep physiology influences these changes.

### Learning incorporates successor representations

Participants performed well in both the Memory Arena task (median accuracy ± MAD: pre-sleep test: 83.6 ± 16.9%; post-sleep test: 73.4 ± 20.7%; *t*(25) = 3.33, *p* = 0.002, Fig 1g) and the perceptual task (median RT ± MAD to target detections: pre-learning: 0.565 ± 0.081 s; post-learning: 0.558 ± 0.093 s; *t*(25) = −0.52, *p* = 0.604).

**Fig 2:**
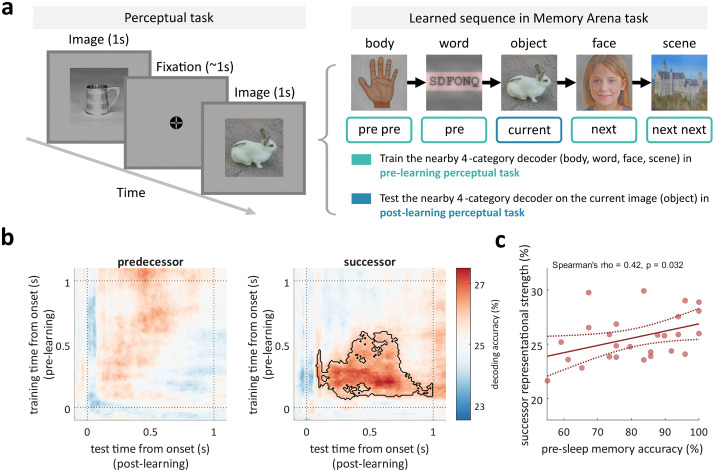
Learning incorporates successor representations. **(a)** Left: example trials from the perceptual task, including image presentation (1,000 ms) and fixation period (750-1,250 ms). Right: decoding approach for the currently displayed image (bunny). Example image sequence from the Memory Arena task (from previous previous, previous, current, next, to next next around the “bunny” image). Decoders were trained on EEG data from the pre-learning perceptual task using the four nearby categories (body parts, letter strings, faces, and scenes) and were tested on EEG data of the “bunny” (object) image in the post-learning perceptual task. Stimuli have been replaced with materials from open-source resources (https://commons.wikimedia.org, https://openclipart.org/) and an AI-generated face dataset (https://github.com/RichardErkhov/ai_generated_faces), used under the MIT License; **(b)** Group-level time-by-time decoding accuracies of predecessor and successor images across participants. Black contour indicates significantly higher decoding accuracy than chance level (*p*_cluster_ = 0.028, *N* = 26, one-tailed cluster-based permutation test); **(c)** Correlation between immediate post-learning memory accuracy and mean successor decoding accuracy within the cluster time window that exhibited above-chance decoding accuracy. The solid line shows the best-fit linear regression line. The dotted lines indicate the 95% confidence bounds for the fitted line. The data underlying this panel can be found in S1 Data.

To examine whether sequence learning reshapes neural representations of visual experiences, we first assessed how well predecessor and successor information could be decoded from neural activity (Fig 2a). Specifically, for each image category (e.g., objects), we trained decoders on EEG data from the pre-learning perceptual task using the remaining four categories (body parts, letter strings, faces, and scenes). We then applied these decoders to EEG signals during the post-learning perceptual task to generate a time-by-time matrix of classifier category prediction. This allowed us to assess the evidence for the immediate predecessor or successor image against the chance level (25%). Results revealed that category decoding accuracy of successor images was significantly above chance (*p*_cluster_ = 0.016, one-tailed cluster-based permutation test; Fig 2b). In contrast, the decoding accuracies for predecessor, second predecessor, and second successor images did not exceed chance levels (all *p*_cluster_ > 0.241; Figs 2b and S1a). Importantly, applying the same decoders to the interference sequence and pre-learning perceptual task revealed no significant effects (*p*_cluster_ > 0.127). In fact, directly comparing successor decoding accuracies between pre- and post-learning perceptual tasks revealed a significant cluster with higher accuracies in the post-learning session (*p*_cluster_ = 0.044, one-tailed cluster-based permutation test; S1b Fig). These results rule out the possibility that the successor representation resulted from perceptual similarities among particular image categories.

Next, we examined whether the strength of successor representation was linked to learning behavior. We first extracted the mean successor decoding accuracy within the cluster time window that exhibited above-chance decoding accuracy for each participant and correlated it with their sequence learning accuracy. We found that greater immediate post-learning memory accuracy was associated with greater successor representational strength in the final perceptual task (Spearman’s rho = 0.42, *p* = 0.032; Fig 2c). In contrast, neither delayed post-sleep memory accuracy nor interference sequence accuracy showed a significant correlation with successor decoding accuracy (*ps* > 0.278; S2b Fig).

Together, these findings suggest that learning image sequences incorporates successor representations, even when such information is not relevant to the current (perceptual) task. Moreover, better immediate post-learning memory performance predicted stronger successor representations. However, delayed post-sleep memory performance was not significantly associated with successor representations, arguing against active sequence recall during the final perceptual task driving the effect.

### Successor representations shift towards high-level visual information

**Fig 3:**
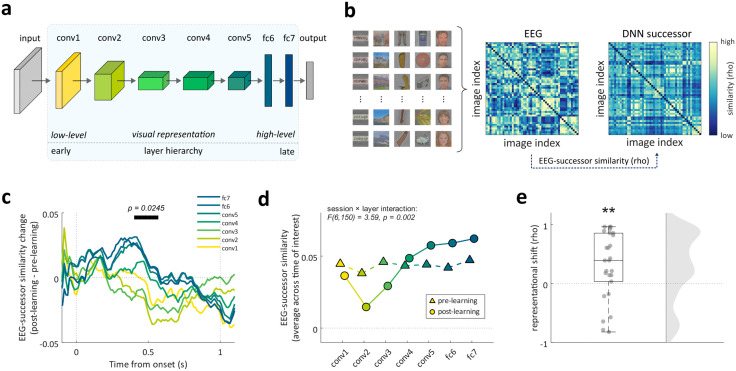
Successor representations shift towards higher-level visual representations. **(a)** Architecture of Alexnet [28]. Hidden layers contain conv1-conv5 and fc6-fc7; **(b)** Illustration of EEG-successor similarity analysis. Left: example images used in the Memory Arena task. Middle: Example EEG similarity matrix. Right: Example DNN similarity matrix of successor images in DNN layer 7 (‘fc7’). Stimuli have been replaced with materials from open-source resources (https://commons.wikimedia.org, https://openclipart.org/) and an AI-generated face dataset (https://github.com/RichardErkhov/ai_generated_faces), used under the MIT License; **(c)** EEG-successor similarity change from pre- to post-learning perceptual tasks across time. Horizontal line indicates a significant interaction cluster between session (pre-/post-learning perceptual task) and layer (conv1–conv5, fc6, fc7) in time (*p*_cluster_ = 0.024, N = 26, cluster permutation tests); **(d)** Group-level EEG-successor similarity during pre-learning and post-learning perceptual tasks within the cluster time window that exhibited a significant session × layer interaction; **(e)** Representational shift: Spearman’s correlations between averaged EEG-successor similarity change (post minus pre) and layer hierarchies. Correlations were Fisher z-transformed for statistical testing. ** indicates *p* < 0.01, *N* = 26, two-tailed one-sample *t t*est. The data underlying this panel can be found in S1 Data.

What is the representational make-up of successor information? To address this question, we applied RSA [7] to compare neural representations with those of a deep neural network (DNN, specifically Alexnet; [28]. This DNN consisted of seven hidden layers (Fig 3a), with earlier layers primarily encoding low-level visual features (e.g., color, contrast), and deeper layers encoding more abstract, higher-level properties (e.g., shape, object identity; [29–32]. We first constructed an image-by-image EEG similarity matrix at each time point, based on pairwise correlations between neural activity patterns elicited during stimulus processing. This was done for both the pre-learning and post-learning perceptual tasks (see Methods; Fig 3b), and only images remembered in both the pre- and post-sleep memory tests were included in this analysis. These matrices capture the time-resolved representational geometry of visual stimuli in the brain. In parallel, we derived model-based pairwise similarity matrices for successor images based on the learned Memory Arena sequence. This was done separately for each layer of the pretrained DNN, spanning a hierarchy from low- to high-level visual representations.

Confirming the sensitivity of these analyses, we found that both EEG and DNN carried category-specific information, with higher within-category similarity than between-category similarity (S3 Fig). To assess the level at which the brain encoded successor information, we correlated neural similarity matrices with DNN-derived similarity matrices at each layer. We hypothesized that neural representations of successor images in the post-learning perceptual task would consist more strongly of higher-level than lower-level DNN features, reflecting a shift toward more abstract representational formats. To test the interaction between session (pre-/post-learning perceptual task) and layer (conv1–conv5, fc6, fc7), we performed a two-way repeated-measures ANOVA across time, applying cluster-based permutation tests as described above. Results showed a cluster with a significant session × layer interaction peaking around 500 ms after image onset (*p*_cluster_ = 0.024; Fig 3c). Within the significant cluster, we found a significant interaction term (*p* = 0.002, Fig 3d) and a main effect of layer (*p* = 0.010) but no main effect of session (*p* = 0.980). Decomposition of the interaction showed a trend for a linear component (*p* = 0.050) and a significant cosine-shaped component (*p* = 0.040), indicating that the changes across layers are best described as a mixture of monotonic and non-linear (curved) changes.

To derive an estimate of this representational shift for each participant, we averaged their layer-specific changes in EEG-successor similarities from pre- to post-learning perceptual tasks within the significant cluster time window and correlated them with layer hierarchy (ranging from 1 to 7) (‘representational shift’ analysis; Figs 3d and S4a). In keeping with the ANOVA result, we found that these correlation values were significantly greater than zero (*t*(25) = 2.88, *p* = 0.007; Fig 3e). In other words, the change in successor representation format after learning followed the progression from superficial to deep layers in a DNN. A time-resolved analysis using this correlational approach likewise converged with the ANOVA results (S4b Fig).

Lastly, we conducted the same analysis using the current (instead of the successor) image. All DNN layers showed significant correlations with EEG activity (all *p*_cluster_ < 0.001; S5a and S5b Fig). Then we averaged similarity values across the 1-second image presentation window for each layer and performed a two-way (session × layer) repeated-measures ANOVA. Results revealed a significant main effect of layer (*F*(6, 150) = 39.10, *p* < 0.001; S5c Fig) but no significant main effect of session (*F*(1, 25) = 0.36, *p* = 0.552). Interestingly, in both the pre- and post-learning perceptual tasks, we observed that while conv1 layer showed lowest correlation with EEG activity, correlation strengths gradually declined from layers conv2 to fc7. To test this decline trend statistically, for each participant, we correlated the similarity values of the six deeper layers (conv2–fc7) with the layer hierarchy (ranging from 2 to 7). This revealed a significant negative correlation (*t*(25) = −2.24, *p* = 0.033; S5c Fig), indicating *t*hat lower-level visual representations of a DNN were more strongly represented in the EEG data than higher-level representations. This pattern likely reflects the perceptual demands of the task, which required visual discrimination (1-back task, Fig 2a). However, in contrast to successor images, no significant interaction between session and layer was found for currently displayed images (*F*(6, 150) = 0.40, *p* = 0.879; S5d and S5f Fig). To further test the interaction between image types (current/successor), session (pre-/post-learning perceptual task), and layer (conv1–conv5, fc6, fc7), we performed a three-way repeated-measures ANOVA, which showed a significant three-way interaction (*F*(6, 150) = 2.64, *p* = 0.018; S5e Fig).

In sum, these findings suggest that learning reshapes successor representations and that these representations take on higher-level visual formats, while the sensory encoding of current images emphasizes lower-level features and remains largely unaffected by learning.

### SO–spindle coupling predicts successor strength and representational shift

**Fig 4:**
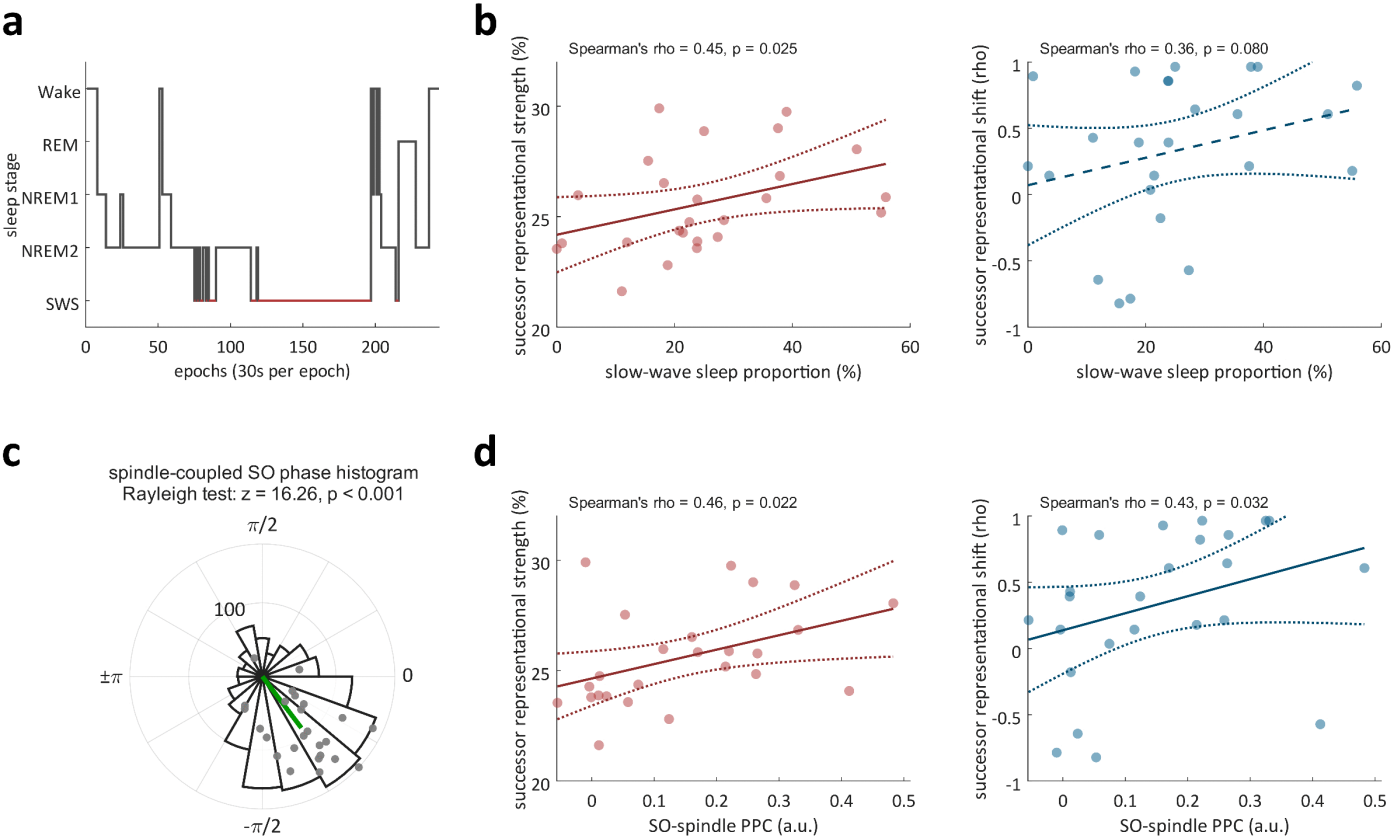
SO–spindle coupling predicts successor representational strength and shift. **(a)** Example sleep hypnogram of one participant. Red lines highlight slow-wave sleep (SWS); **(b)** Correlation between SWS proportions and successor representational strength (left) and shift (right) across participants within the previously identified significant time windows; **(c)** Circular histogram of preferred SO phase angles pooled across all participants. Each dot represents one participant’s phase vector (angle and mean vector length). The green line marks the mean phase vector across participants, with 0 corresponding to the SO up-state and ±π to the down-state. Statistical significance was assessed using the Rayleigh test for non-uniformity of phase distributions across participants; **(d)** Correlation between PPC values at Cz and successor representational strength (left) and shift (right) across participants within the previously identified significant time windows. The solid/dashed line in panels b and d shows the best-fit linear regression line. The dotted lines indicate the 95% confidence bounds for the fitted line. Each dot represents one participant. *N* = 25.

Lastly, we examined how sleep physiology may contribute to incorporating successor representations after learning. Nap EEG data were scored with automatic algorithms and manually validated (Fig 4a; see Methods). One participant who exhibited only NREM 1 sleep was excluded, resulting in a final sample of 25. We then derived the proportion of SWS for each participant (median ± MAD: 23.7 ± 11.37%; S6a Fig). These SWS proportions were then correlated with successor representational strength (i.e., decoding accuracy) and shift averaged within the previously identified significant time windows. Higher proportions of SWS were associated with greater successor representational strength across participants (Spearman’s rho = 0.45, *p* = 0.025, Fig 4b) and with a trend for towards greater representational shift to high-level formats (Spearman’s rho = 0.36, *p* = 0.080, Fig 4c). Importantly, no significant effects were found for any other sleep stage (all *p* > 0.158).

We then examined whether the link between sleep and successor incorporation might be mediated by the coupling of key NREM sleep signatures, i.e., SOs and spindles [23,24]. Using an established detection protocol [33], slow oscillations and spindles were identified separately, and their coupling was defined as the spindle envelope maximum occurring within the onset and offset of a slow oscillation. Across participants, we observed an average of 50.9 ± 19.9 coupled SO–spindle events. To quantify coupling strength, we computed pairwise phase consistency (PPC) at Cz for each participant using all SO phases occurring at the spindle envelope maxima (see Methods and Fig 4b). PPC values were then correlated with successor representational strength and shift. We found that SO–spindle coupling was associated with stronger successor representations (Spearman’s rho = 0.45, *p* = 0.022, Fig 4e) and a larger representational shift toward higher-level formats (Spearman’s rho = 0.41, *p* = 0.032, Fig 4f). Additional analyses ruled out the possibility that these findings were driven by correlations between pre-sleep memory performance and SWS/PPC (*p*s > 0.102) or by interdependence between successor representational strength and shift (Spearman’s rho = 0.14, *p* = 0.500).

## Discussion

In this study, we tracked changes in representational geometry before and after learning a spatio-temporal image sequence (Fig 1). By combining high-density EEG recordings and multivariate decoding methods, we found that learning a temporal sequence leads to the incorporation of successor representations in a subsequent non-memory task. Specifically, after learning, the neural activation profiles while viewing an image contained category information about its immediate sequence successor, even though sequence information was irrelevant for the current (perceptual) task. Individuals with better pre-sleep memory performance exhibited greater levels of successor integration (Fig 2). Comparing the representational format of successor information with different layers of a DNN suggested that low-level visual features before learning gave way to higher-level, abstract visual properties after learning (Fig 3). Crucially, both the strength and shift in format of successor representations were associated with the strength of SO–spindle coupling in a post-learning nap (Fig 4). Together, these findings suggest that sequence learning incorporates high-level successor representations, and that specific NREM sleep physiology facilitates this process.

Our findings extend the theoretical framework of successor representations, which were originally proposed in the context of reinforcement learning to integrate current and anticipated future states [1]. More recent work has suggested that this framework may also apply to episodic memory [2,3,34]. In line with this view, our results showed robust category encoding of successor (but not predecessor) images after sequence learning, even in a task that did not require the retrieval of temporal structure (Fig 2b). Notably, this forward-leaning pattern echoes prior evidence for temporal asymmetry in the medial temporal lobe, where learning induced stronger forward than backward predictive structure [13]. Furthermore, the strength of successor representations correlated with immediate recall performance (after learning and before sleep; Fig 2c), linking behavioral expressions of sequence learning efficacy to the phenomenon of successor incorporation. The tendency to integrate successive experiences is also consistent with longstanding models of temporal organization in episodic memory. For instance, the temporal context model [35,36] proposes that memory retrieval is guided by a context signal that evolves over time, favoring the recall of temporally subsequent items. Recent computational work has integrated this model with successor representation theory to better capture the predictive structure of memory-guided behavior [6]. Complementing these behavioral and computational accounts, recent human intracranial EEG studies have shown that neurons in the medial temporal lobe encode the temporal structure of visual experiences [4,5,37]. Although these studies have not distinguished between forward- and backward adjacency, our findings raise the possibility that these temporal codes may primarily reflect forward (successor) information.

Crucially, the successor representations observed here persisted despite the introduction of an interference sequence in which the same images were re-encoded in a different category order before the post-sleep test. We found no evidence for successor representations based on the interference sequence (S1b Fig), nor did behavioral performance for that sequence affect the strength of successor representations for the main learned sequence (S2b Fig). This demonstrates that the predictive structure established during initial learning (and followed by sleep) remains stable and is not trivially re-instantiated by recent exposure. Future work could systematically assess the effect of different levels of interfering sequence information on final successor representations.

To better understand the format of successor representations, we resorted to tools commonly used in vision neuroscience. Specifically, over the past decade, advances in convolutional DNNs in the domain of computer vision (e.g., Alexnet; [28]) and natural language processing (e.g., GPT-2; [38]) have provided powerful tools for understanding how the brain organizes complex information. After training, these DNNs form hierarchical representations that progressively transition from low-level sensory features to increasingly abstract, high-level properties, paralleling the structure of human cortical processing [29–32). Using RSA [7], we assessed the alignment of neural activity with different latent layers of Alexnet. Our results revealed a shift in successor representational format: after learning, neural activity patterns became less aligned with low-level visual features and more aligned with high-level, abstract properties (Fig 3c–3e). These findings suggest that learning not only incorporates successor representation but also reorganizes it into a more conceptual format. The observed shift of successor representation complements and extends prior intracranial EEG studies, which revealed a transformation of currently displayed image representations from perceptual to conceptual formats with repeated exposure in short- as well as long-term memory tasks [39–41]. It further aligns with theories proposing abstraction as a key feature of memory consolidation [42–47].

Intriguingly, we found that the learning-induced changes in neural representations were modulated by sleep physiology. First, higher proportions of SWS during a post-learning nap predicted stronger successor representations, though the association with representational shift to high-level formats only showed a statistical trend (Fig 4b). Importantly, investigating the oscillatory mechanisms of NREM sleep and assessing the coupling of SOs and spindles, we found that coupling strength predicted both the strength of successor representations and the extent of their abstraction (Fig 4d). These findings align with prior evidence that SO–spindle coupling serves as a key neurophysiological marker for memory reactivation [21] and provides a critical time window for hippocampal ripple activity [22,23], processes that are closely linked to neural replay and the reorganization of representational structures [15,16]. It deserves mention that our use of a learning-to-criterion procedure with repeated forward testing may have selectively strengthened forward temporal associations, potentially favoring successor representations. However, a study by Drosopoulos and colleagues [48] found that, compared with wake, sleep selectively enhances forward, but not backward, associative links in word sequences, even for items that were never forward-tested during initial learning. This indicates that consolidation during sleep might preferentially strengthen the successive temporal structure of experience. Future work could use different learning criteria and learning procedures with minimal directional testing to test this hypothesis more directly.

Although our study did not include a wake control group, recent work offers compelling support for a sleep-specific effect. Diamond and colleagues [24] found that participants who slept after real-life sequential learning (a guided art tour) exhibited enhanced temporal order memory but a loss of low-level perceptual detail in behavior—an effect that was specifically linked to SO–spindle coupling. This pattern closely parallels our representational-level findings, suggesting that SO–spindle coupling may bias memory consolidation towards abstract, temporally structured information. Relatedly, recent MEG work using a sequential planning paradigm reported that consolidation promotes successor representation-like forward “rollout” signals in the brain [49]. Together, these results converge with our data suggesting that successor representations are shaped by sleep-dependent consolidation.

Beyond episodic memory, our work also resonates with findings from sequence learning in other domains. For example, in the motor domain, sleep has consistently been shown to support the consolidation of sequential skills, with performance improvements typically emerging after sleep compared to equivalent wake intervals [50–53]. Also, motor sequence memory reactivation has been observed time-locked to coupled SO–spindle complexes, specifically in regions critical for motor sequence learning [54]. A similar sleep-related effect has also been observed in prospective memory, where sleep enhances the ability to remember future intentions [55] . These findings, together with our current results, converge to the notion that specific NREM sleep characteristics facilitate the formation of predictive associations across different domains.

More broadly, our findings shed light on how the brain updates its internal model of the world. Successor representations provide a mechanism by which experience can reshape predictive structures, allowing individuals to anticipate future events based on learned regularities. This predictive capacity is crucial in everyday contexts where behavior unfolds over time. An open question in our study, however, concerns the longevity of these representations. Do successor representations persist over time, or do they eventually “wash out” in the absence of repeated/continued learning? Investigating the durability and stability of these effects over longer timescales remains an important direction for future work.

Several considerations should be taken into account when interpreting the present findings. First, although our sample size was in line with previous EEG and sleep studies, it is modest by current standards [56]. The correlational results should therefore be interpreted with caution. Second, the category-level decoding of successor information, in line with the representational shift toward higher-level abstract features, raises the question of whether the observed successor representations reflect specific image identities or a broader category-based schema, and whether they extend beyond the immediate one-step successor. While previous studies have demonstrated item-level successor representations [5,4] as well as multi-step predictive structure [49], addressing these questions will require future work using designs that permit item-level resolution alongside analytical approaches sensitive to distance-dependent dynamics. Additionally, future work should test whether successor representations generalize to novel exemplars from the same categories, which would provide stronger evidence for abstraction beyond the specific trained images. Third, post-learning wakefulness could likewise be a crucial factor driving such integration and transformation, for instance during periods of quiet rest that may allow ongoing replay and reorganization of newly encoded experiences [57,58]. Future work could include post-learning wakeful rest control groups to more clearly dissociate wake- and sleep-related contributions to representational reorganization.

In conclusion, our findings show that sequential learning induces successor representations in the human brain, even in a perceptual task unrelated to sequential information. These representations shift from perceptual to abstract formats and are supported by post-learning sleep physiology, particularly SO–spindle coupling. Together, these results advance our understanding of how the human brain integrates temporal structure into our internal world model.

## Methods

### Participants

Thirty healthy adults (9 males; mean age = 25 years; range = 19–39 years) participated in the study. Our sample size was determined in accordance with previous human sleep and memory studies (*N* = 30 in [59]; *N* = 35 in [60]; *N* = 24 in [61]; *N* = 25 in [22]). Participants received either course credit or monetary compensation for their participation. To ensure sleep-impairing factors did not confound the results, participants were screened for the following exclusion criteria: engagement in night shift work within the past year, recent travel across time zones (within the past two weeks), current use of medications affecting sleep, any history of neurological, psychiatric, or sleep disorders and regular consumption of more than one cigarette per day. Behavioral performance in the Memory Arena task was quantified using two metrics: pairwise sequential accuracy (percentage of correctly ordered adjacent image pairs) and placement error (in pixels). Participants were classified as outliers if their performance on either metric exceeded three scaled median absolute deviations from the group median in both the final learning block and the pre-sleep memory test, as determined using MATLAB’s *isoutlier* function (S1 Table). During the final learning block, two participants showed extreme values on both sequence accuracy and placement error, one showed extreme sequence accuracy only, and one showed extreme placement error only. During the pre-sleep test, two of these participants again showed extreme placement error. To ensure a sufficient number of remembered image pairs for robust representational similarity analyses and to include only participants who had learned the full spatio-temporal sequence, these four participants were excluded, yielding a final sample of 26 participants for the reported analyses. All participants provided written informed consent, and the study was approved by the University of Oxford’s ethics committee (approval code: R85832/RE001). The study has been conducted according to the principles expressed in the Declaration of Helsinki. The learning data from the Memory Arena task have been reported previously in Büchel and colleagues [26].

### Experimental design

Participants first performed a perceptual task including 50 unique images, followed by the ‘Memory Arena’ task presenting the same images in fixed temporal and spatial sequences ([26]; for an earlier version of the task, see also [27]. After reaching a learning criterion, participants took a ~2-hour nap. Memory accuracy was tested before and after sleep, followed by a repetition of the perceptual task. EEG recordings were applied throughout the experiment.

The perceptual task included 50 images from five categories (objects, faces, scenes, letter strings, and body parts). Each main category comprised two subcategories: Body parts (upper body, lower body), Face (young girl, old man), Object (animate, inanimate), Scene (natural, unnatural), and Letter strings (capitalized pseudowords, uncapitalized pseudowords). Each main category contained 10 images, and each subcategory contained five. During the perceptual task, each image was presented for 1,000 ms, followed by a fixation period lasting 750–1,250 ms. Participants performed a one-back repetition detection task, pressing the ‘down arrow’ key whenever an image was repeated. Each session comprised 660 trials (including 10% repeated target trials), and participants performed the task with high accuracy (mean ± SD: 98.32 ± 0.74%). For subsequent analyses, only correct trials involving non-repeated images were included. Importantly, the image presentation order in the two perceptual task sessions was pseudo-randomized and unrelated to the sequences learned in the Memory Arena task, enabling us to isolate the effects of sequence learning on visual brain representations.

In the Memory Arena task, participants learned the sequential and spatial structure of 50 images across repeated learning cycles (Fig 1, detailed in [26]. These 50 images were organized into 10 subsequences of five images each, following one of two fixed category orders: (i) letter string, scene, object, face, or (ii) object, scene, letter string, face, with body part images randomly inserted to obscure the primary category sequences. The two subsequence types were counterbalanced across participants. Each learning cycle consisted of two exposure blocks, where images were presented sequentially at their respective locations, followed by a test block in which participants reconstructed the sequence and spatial layout. Each image was presented twice per learning cycle (once per exposure block). Note that spatial components and temporal components are independent in the design because knowing an image’s spatial location does not in itself specify its temporal position within the sequence. Learning continued until participants reached at least 66% accuracy or a maximum of 60 min had elapsed. Importantly, learning criterion and trial-by-trial feedback were based on temporal-order accuracy quantified as correctly ordered adjacent image pairs. The 66% memory threshold was chosen to avoid ceiling effects and to maintain an intermediate level of memory strength, as this range has previously been shown to be sensitive to sleep-related consolidation [27]. On average, participants reached the criterion by testing block 3, i.e., after 6 exposure blocks, which means, on average, each image was viewed 6 times during initial learning.

Following the learning phase, participants performed a 5-min attention task. They were instructed to fixate on a central cross and count each instance it turned dark gray, while ignoring instances where it turned light gray. After that, participants completed the same memory test as in the Memory Arena task, which served as an assessment of pre-sleep memory performance. They were then given a 2-hour nap opportunity (mean sleep duration ± SD: 71 ± 28 min), during which polysomnographic data were recorded to monitor sleep stages. Upon waking, participants engaged in an interference task. In this phase, they re-encoded the same set of 50 images, but with both the sequence and spatial locations altered. Specifically, the images were arranged into different subsequences that followed an alternative fixed category order (median sequence accuracy ± MAD: 16.3 ± 12.7%). Also, each image’s new location differed from its original location by at least 5 pixels (Euclidean distance). The encoding procedure for this new configuration mirrored that of the initial learning phase, but consisted of only one round. Following the interference encoding, participants were asked to recall the new sequence and spatial arrangement. Next, they completed a memory test to retrieve the originally learned sequence and layout, which served as an assessment of post-sleep memory performance. To quantify potential intrusion, we rescored the post-sleep test using interference-sequence labels; performance was near floor (median ± MAD: 2.04 ± 3.02%), compared to robust performance when scored with the main-sequence labels (73.4 ± 20.7%), indicating minimal behavioral confusion between sequences. The post-sleep assessment was followed by a final repetition of the perceptual task.

### EEG recording and preprocessing

EEG data were recorded using a 64-channel Brain Products system at a sampling rate of 500 Hz. Electrodes were positioned according to the international 10–20 system, with FCz as the recording reference and AFz as the ground. Six electrodes were reassigned for auxiliary recordings: two for mastoids, two for electromyography, and two for electrooculography. This configuration left 58 channels available for scalp EEG recording. Our analyses focused on the EEG data collected during the perceptual task sessions.

Preprocessing was conducted using the following procedures: First, data were downsampled to 250 Hz and high-pass filtered at 0.1 Hz. Line noise in the 49–51 Hz range was removed, and data were re-referenced to the common average. Channels identified as noisy during visual inspection were interpolated; in total, 11 bad channels were replaced across 26 participants.

The filtered EEG signal was then segmented into epochs ranging from 500 ms before to 1,500 ms after image onset. Independent component analysis was performed on these epochs to identify and remove components reflecting eye blinks, based on visual inspection. The cleaned data were smoothed using a 200 ms moving mean window (MATLAB’s *smoothdata* function), baseline corrected using the 200 ms pre-stimulus interval, and finally z-scored across trials using MATLAB’s *normalize* function.

### Successor representation decoding

To investigate whether sequence learning changes visual brain representations, we conducted multivariate pattern classification analyses using EEG voltage signals from all channels. Within each participant, we first averaged the EEG signal in sliding time windows of 20 ms (five data points) with a step size of 12 ms (three data points). The resulting voltage patterns across all channels served as input features for a multiclass linear discriminant analysis, implemented using the MVPA-Light toolbox [62].

To assess whether learning induced representations that incorporated sequential structure—such as successors—we examined how well neural activity during post-learning perceptual task trials reflected category information from nearby positions in the learned Memory Arena sequence. For each category (e.g., objects), we trained the classifier using EEG data from the other four categories (e.g., body parts, letter strings, faces, and scenes) in the pre-learning perceptual task session and tested it on untrained object images in the post-learning perceptual task session. This cross-session generalization design allowed us to identify changes in neural representations that emerged specifically as a result of learning, while minimizing concerns of overfitting or confounding temporal proximity effects. We then compared the predicted category label with the category of neighboring items in the sequence participants had learned during the Memory Arena task. For instance, if participants had learned a subsequence such as “letter string → object → face,” we examined whether EEG patterns during object trials were more likely to be classified as the successor category (face) or the predecessor category (letter string). This procedure yielded an accuracy estimate for each time point that the decoded category matched the immediate predecessor or successor in the learned sequence. Decoding accuracy was calculated for each image trial and for each sequence position within the 50-image sequence. These accuracies were then averaged across trials to generate a time-by-time matrix of classifier predictions, which were then compared against the chance level (25%) using cluster-based permutation statistical tests (see Statistical analysis). We also extended this analysis to more distal sequence positions—examining decoding accuracies for the second predecessor and successor—to further probe the spread of sequence-related representations (S1a Fig).

To relate successor representation to memory performance, we correlated decoding accuracies with pre- or post-sleep sequential memory accuracies across participants at each training and test time point. Sequential memory accuracy was indexed by the proportion of correctly ordered image pairs, in line with our companion work, where learning rate based on pairwise sequence accuracy correlated with sequence information in EEG and eye movements during learning [26].

Lastly, to confirm that particular effects emerged as a consequence of learning, we applied the same decoding procedures to pre-learning perceptual task data. Successor decoding accuracies from the pre-learning perceptual task session were then compared to those from the post-learning perceptual task session using the same statistical approach. The absence of sequence-based classification patterns prior to learning, along with a significant increase in successor decoding after learning, served as evidence that the observed representational changes were specifically induced by sequence learning occurring in between (S1c Fig).

### Representational similarity analysis

To investigate the format of the successor information, we compared neural representations with those derived from a DNN (Alexnet; [28]. Alexnet is pretrained on the ImageNet dataset to perform object classification, and has been shown to exhibit a hierarchical organization of visual representations similar to the ventral visual stream in humans and other primates [29–32]. Specifically, earlier layers (e.g., conv1–conv3) predominantly encode low-level features such as edges and textures, whereas deeper layers (e.g., fc6–fc7) capture increasingly abstract and categorical information [32].

For each image used in the experiment, we extracted activation patterns (expressed as feature vectors) from each layer of Alexnet. These patterns were used to construct representational similarity matrices by computing pairwise Spearman’s correlations between feature vectors across images. Two types of DNN similarity matrices were created:

1. DNN current similarity matrices: computed using the features of the currently displayed image.
2. DNN successor similarity matrices: computed using the features of each image’s successor (i.e., the image that followed in the learned sequence), rather than the image itself.

After deriving DNN similarity matrices, we next computed EEG-based similarity matrices. For each participant, we selected images whose successors were correctly identified in both memory tests, ensuring reliable recall. Using these remembered images, we computed pairwise Spearman’s correlations of EEG voltage patterns across channels, using the same sliding time window approach as in our decoding analyses.

As a quality-control step, we verified that both EEG- and DNN-derived similarity matrices contain reliable category structure (higher within- than between-category similarity). Within-category similarity was calculated by averaging the correlations of all image pairs belonging to the same category. Between-category similarity was computed by averaging the correlations of all image pairs in which the two images belonged to different categories. The category-specificity index was then obtained by subtracting the between-category similarity from the within-category similarity. Both DNN and EEG matrices showed significantly higher within-category similarity, confirming that category-level structure was present (S3 Fig).

We next assessed the correspondence between EEG and DNN representations using the same subset of images for each participant’s analyses. First, we examined the correlation between EEG and the DNN current similarity matrix for each layer (EEG–current similarity). This analysis replicated prior findings showing a strong alignment between EEG activity during perception and DNN-derived representations (S5a and S5b Fig; [39–41]. To evaluate whether EEG activity differentially reflected low- or high-level DNN features, we first averaged, for each participant, EEG–current similarity across the two perceptual task sessions and across the 1-second image presentation window. Then the averaged layer-specific EEG–current similarity was correlated with DNN layer hierarchy (ranks of conv2 to fc7). Positive values indicated stronger alignment with higher-level layers, and negative values indicated alignment with lower-level layers. Correlations were Fisher z-transformed and correlation values equal to ±1 were winsorized to ±0.99 to avoid infinite values during Fisher z-transformation.

To examine how learning influenced neural representations, we assessed the similarity between EEG and DNN successor representations (EEG-successor similarity). Concretely, the EEG similarity matrices are computed from neural responses to the *current* stimulus, whereas the model matrices are constructed from deep-network features of the *successor* stimulus (as defined by the learned sequence). We first examined collinearity between the successor-based and current-image DNN representational similarity matrices and found significant correlations between these two matrices in several layers (conv1, conv4, fc7; *p*s < 0.002). To minimize the influence of current image representations in the EEG data, we regressed out the DNN current matrices from the EEG similarity matrices and correlated the residuals with the DNN successor similarity matrices. This correspondence was compared before and after learning for each DNN layer, using cluster-based permutation tests to correct for multiple comparisons across time. Additionally, to quantify learning-related changes during stimulus presentation, we averaged the similarity differences across the 0–1 s post-onset window.

Finally, to quantify representational shifts across the DNN hierarchy (conv1 to fc7), we correlated the magnitude of EEG–successor similarity changes with layer hierarchy (ranks 1–7). This was done both across the entire time window and for the average within that window. As before, correlations were Fisher z-transformed and winsorized to ±0.99.

### Sleep architecture and its relation to successor representations

Sleep staging for each participant was performed based on 30-second epochs, using EEG polysomnographic recordings in accordance with the American Academy of Sleep Medicine (AASM) guidelines. Stages included wakefulness, NREM1, NREM2, NREM3 (SWS), and REM sleep. Initial automated staging was conducted using two AI-based tools, YASA [63] and Somnobot (https://somnobot.fh-aachen.de). The transitions between stages and any discrepancies between the two automated outputs were then carefully reviewed by two experienced sleep researchers, who finalized the classification of each epoch.

Following staging, we calculated the proportion of time each participant spent in each sleep stage. To examine the relationship between sleep architecture and successor representations, we correlated the proportion of each stage with (i) the successor representational strength (decoding accuracies) obtained from the decoding analysis and (ii) the successor representational shift obtained from the RSA.

### SO–spindle coupling and its relation to successor representations

EEG data recorded during sleep were processed using the same acquisition system and preprocessing pipeline as in the wake task. SOs and spindles were identified based on an established protocol [33]. Specifically, for SO detection, the signal was band-pass filtered between 0.3 and 1.25 Hz. Zero crossings were extracted, and candidate events were defined as segments between two consecutive positive-to-negative crossings. Events lasting 0.8–2 s were retained, and their trough and trough-to-peak amplitudes were computed. Candidates exceeding the mean + 1 s.d. of both amplitude measures were labeled as SOs.

For spindle detection, the signal was band-pass filtered at 12–16 Hz, and the root mean square (RMS) signal was calculated using a 200-ms window and smoothed with the same kernel. A spindle was defined as an interval during which the smoothed RMS exceeded the mean + 1 s.d. for 0.4–3 s, excluding periods above the mean + 9 s.d. in order to remove artifacts. Events were required to contain at least six oscillatory cycles in the raw signal. Onset and offset corresponded to the upward and downward crossings of the RMS threshold; the spindle center was the envelope maximum.

SO–spindle coupling was assessed during NREM sleep, in keeping with prior work [24]. Only spindles whose envelope maximum occurred between an SO onset and offset were considered. To quantify SO–spindle coupling, the EEG signal was filtered in the SO band to extract instantaneous phase (Hilbert transform). For each spindle, we extracted the corresponding SO phase $\left\{\phi_{\text{1}},\phi_{\text{2}},\ldots ,\phi_{N}\right\}$ at the spindle envelope maximum. We focused on electrode Cz because it typically shows robust SO and spindle rates and SO–spindle coupling at Cz has previously been linked to memory reactivation [21]. Coupling strength was indexed using PPC [64], a bias-free metric that is robust to differences in the number of detected events (and associated phase values) across participants. PPC was computed as the average cosine of all pairwise phase differences:


$$\text{PPC}=\;\frac{\text{1}}{N(N-\text{1})}\sum_{\begin{array}{c} i,j=\text{1}\\ i\neq j \end{array}}^{N}\text{cos}(\phi_{i}-\phi_{j})\mathrm{\backslash ]}$$

This measure quantifies the consistency of phase alignment while remaining independent of event count. Higher PPC values indicate stronger phase locking between slow oscillations and spindles.

Finally, to examine the relationship between SO–spindle coupling and successor representations, we correlated the PPC values with (i) the successor representational strength (decoding accuracies) obtained from the decoding analysis and (ii) the successor representational shift obtained from the RSA. We also extended this analysis from Cz to all EEG channels to derive topographical patterns, which revealed a focal cluster centered around Cz (S6d Fig).

### Statistical analysis

To assess the temporal profile of successor representations, we used cluster-based permutation testing (implemented in FieldTrip; [65] to correct for multiple comparisons across time in both decoding and representational similarity analyses. This nonparametric approach identifies clusters of consecutive time points that exceed a predefined threshold (here: *p* < .05), with cluster-level significance evaluated through 2,000 random permutations of the data. For decoding analyses, we used one-tailed paired *t*-*t*ests, as only above-chance effects were theoretically meaningful. For representational similarity analyses, we used *F*-tests to test the interaction between session (pre-/post-learning) and layer hierarchy (from conv1 to conv5, fc6, fc7), and two-tailed *t*-*t*ests to test whether representational shift from early layers to deep layers (rho) differed from zero. Significant clusters from the decoding and representational similarity analyses defined the time windows of interest. The mean values within these windows were then correlated with SWS proportions and SO–spindle coupling (PPC) using two-tailed Spearman correlation.

## Supporting information

S1 Data Excel spreadsheet with individual numerical data organized into separate sheets corresponding to the following figures and figure panels: 1g, 3e, S3d, and S6a. (XLSX)

S1 Table Behavior of group median and outliers in final learning block and pre-sleep test. *Note.* Values exceeding more than three-scaled median absolute deviation (MAD) from the group median were highlighted in bold.
(XLSX)

S1 Fig Decoding results of second predecessor and successor. **(a)** Group-level time-by-time category decoding accuracies of second predecessor (left) and second successor (right) images across participants (*N* = 26). Decoders were trained in each time point in the pre-learning perceptual task and were then tested in the post-learning perceptual task; **(b)** Group-level time-by-time category decoding accuracies of successor images with respect to the interference sequence. No significant cluster was found (*p*_cluster_ > 0.124, *N* = 26, one-tailed cluster permutation tests); **(c)** Comparison of successor decoding accuracies between the pre- and post-learning sessions. Black contour indicates significantly higher correlations than zero (*p*_cluster_ = 0.044, *N* = 26, one-tailed cluster permutation tests).
(TIF)

S2 Fig Correlation between successor representational strength and behavior. **(a)** Correlation between post-sleep memory accuracy and mean successor decoding accuracy within the cluster time window that exhibited above-chance successor decoding in the main learned sequence (*N* = 26); **(b)** Correlation between interference sequence accuracy and mean successor decoding accuracy within the cluster time window (*N* = 26). Each dot represents one participant.
(TIF)

S3 Fig Category representation in EEG and DNN. **(a)** Average image-image neural representational similarity matrix across 1-s image presentation window (*N* = 26) during the pre-learning perceptual task; **(b)** Neural category-specific information (within-category similarity minus between-category similarity) for the five categories across time during the pre-learning perceptual task (all *p*s < 0.001, *N* = 26, two-tailed *t*-tests); **(c)** Neural category-specific information in the perceptual task (averaged across the five image categories). Shaded areas indicate standard error across participants. Horizontal lines indicate significant clusters in time (*p*_cluster_ < 0.001, *N* = 26, two-tailed cluster permutation tests); **(d)** DNN category-specific information across hidden layers. Error bars indicate standard error across categories (*n* = 5). The data underlying this panel can be found in S1 Data.
(TIF)

S4 Fig Representational changes between EEG and DNN for successor images. **(a)** Individual participant changes in EEG-successor similarity across DNN layers, averaged across the 0–1 s time window. Each line represents one participant (from yellow to blue: conv1, conv2, conv3, conv4, conv5, fc6, fc7); **(b)** Time-resolved correlation between layer hierarchy and representational changes. Shading indicates standard error across participants. Correlations were Fisher z-transformed for statistical testing, with extreme values (>0.99 or <−0.99) winsorized. Horizontal line indicates significant clusters in time (*p*_cluster_ = 0.018, *N* = 26, two-tailed cluster permutation tests).
(TIF)

S5 Fig Representational similarity analyses between EEG and DNN for currently displayed images. **(a)** Spearman’s correlations (EEG-current similarity) between EEG similarity matrix in the pre-learning session and DNN current similarity matrices of each layer (from yellow to blue: conv1, conv2, conv3, conv4, conv5, fc6, fc7). Horizontal lines indicate significant correlations (*p*_cluster_ < 0.001, *N* = 26, two-tailed cluster permutation tests); **(b)** EEG-current similarity in the post-learning session (*p*_cluster_ < 0.001, *N* = 26, two-tailed cluster permutation tests); **(c)** Average EEG-current similarity across the two sessions. Bar plot shows mean similarity for each DNN layer in the 0-1s time window. Error bars indicate standard error across participants. Inset plot shows the correlations between average similarity and layer hierarchy (from conv2 to fc7). Correlations were Fisher z-transformed for statistical testing, with extreme values (>0.99 or <−0.99) winsorized. * indicates significant difference from zero (*p* = 0.033, two-tailed *t* test); **(d)** EEG-current similari*t*y change from pre- to post-learning perceptual sessions across time. No significant interaction was found between the session (pre/post-learning) and layer hierarchy (*p*_cluster_ > 0.879, *N* = 26, *F* value cluster permutation tests); **(e)** Average EEG-current/successor similarity change within the predefined significant time window. Significant interaction was found between the image types (current/successor), session, and layer hierarchy (*p* = 0.018, *N* = 26, 3-way repeated measures ANOVA); **(f)** Time-resolved correlation between layer hierarchy and similarity changes (representational shift). Shaded areas indicate standard error across participants. No significant difference was found between correlation values and zero (*p*_cluster_ > 0.800, *N* = 26, two-tailed cluster permutation tests).
(TIF)

S6 Fig Correlations between successor representational changes and the proportions of sleep stages. **(a)** The proportions of all sleep stages during an approximately 2-hour nap across participants. Each dot represents a participant. The data underlying this panel can be found in S1 Data; **(b)** Topography of correlation between SO–spindle PPC and successor representational strength (left; *N* = 25) and shift (right; *N* = 25). White circle indicates Cz electrode.
(TIF)

## Abbreviations

DNN: deep neural network
EEG: electroencephalography
PPC: pairwise phase consistency
RMS: root mean square
RSA: representational similarity analysis
SWS: slow-wave sleep
